# Lung NETs and GEPNETs: One Cancer with Different Origins or Two Distinct Cancers?

**DOI:** 10.3390/cancers16061177

**Published:** 2024-03-17

**Authors:** Georgios Evangelou, Ioannis Vamvakaris, Anastasia Papafili, Maximilian Anagnostakis, Melpomeni Peppa

**Affiliations:** 1 3rd Department of Medicine, National and Kapodistrian University of Athens, Sotiria Chest Diseases Hospital, 11527 Athens, Greece; moly6592@yahoo.com; 2 Department of Pathology, Sotiria Chest Diseases Hospital, 11527 Athens, Greece; i.vamvakaris@yahoo.gr; 3 Department of Oncology, Iatriko Kentro Athinon Marousi, 15125 Athens, Greece; natasa_papafili@yahoo.co.uk; 4 Endocrine Unit, 2nd Propaedeutic Department of Internal Medicine, Research Institute and Diabetes Center, Attikon University Hospital, School of Medicine, National and Kapodistrian University of Athens, 12461 Athens, Greece; molypeppa@med.uoa.gr; 5 3rd Department of Internal Medicine, Sotiria General Hospital, 11527 Athens, Greece

**Keywords:** lung NETs, neuroendocrine tumors, typical carcinoid, atypical carcinoid, pulmonary NETs, EGFR, DLL3, immunotherapy

## Abstract

**Simple Summary:**

This review examined the disparities between lung neuroendocrine tumors and gastroentero-pancreatic neuroendocrine tumors, two types of neuroendocrine tumors that originate from different parts of the body and have historically been treated similarly. This research delves into the differences in genetic makeup, behavior, and response to treatments such as chemotherapy, immunotherapy, and targeted therapies between these two types of tumors. This study aimed to explore these distinctions to develop more personalized and effective treatment strategies for patients with lung and gastroenteropancreatic neuroendocrine tumors. By recognizing and treating these two types of cancer as distinct entities rather than as a single disease, the medical community can significantly improve patient outcomes and highlight the importance of this research.

**Abstract:**

Lung neuroendocrine tumors (LNETs) and gastroenteropancreatic neuroendocrine tumors (GEP-NETs) are two distinct types of neuroendocrine tumors (NETs) that have traditionally been treated as a single entity despite originating from different sources. Although they share certain phenotypic characteristics and the expression of neuroendocrine markers, they exhibit differences in their microenvironment, molecular mutations, and responses to various therapeutic regimens. Recent research has explored the genetic alterations in these tumors, revealing dissimilarities in the frequently mutated genes, the role of EGFR in carcinogenesis, the presence of transcription factors, and the immunogenicity of the tumor and its microenvironment. Spread Through Air Spaces (STAS), a phenomenon unique to lung carcinomas, appears to play a crucial role in LNET prognosis. These distinctions are also evident in the cascade response of lung and GI tract neuroendocrine tumors to somatostatin analogs, Peptide Receptor Radionuclide Therapy (PRRT), chemotherapy, and immunotherapy. Identifying similarities and differences between the two groups may improve our understanding of the underlying mechanisms and facilitate the development of more effective treatment strategies.

## 1. Introduction

The scarcity of occurrence and lack of research data over several decades has resulted in great uncertainty in the management of different scenarios in clinical practice for patients with lung NETs. In the majority of pharmaceutical trials in recent decades, lung NETs have not been included in the patient population sample or are represented in very small proportions compared to gastroenteropancreatic NETs (GEP-NETs); hence, the efficacy of various treatments in this group remains uncertain [1,2,3]. The first edition of the European Society for Medical Oncology (ESMO) guidelines for lung neuroendocrine tumors (LNETs) was introduced in 2012, and the most recent updated version was made available in 2021 [4,5]. The past decade has seen significant research efforts aimed at elucidating various aspects of carcinogenesis and the biological behavior of both lung carcinoids and GEP-NETs, with a focus on similarities and important differences that often result in divergent treatment pathways.

Well-differentiated NETs are classified as low- or intermediate-grade tumors (G1 and G2) according to criteria established by the World Health Organization (WHO) [6]. The G1 and G2 nomenclature is mainly used for NETs of the gastrointestinal tract, mainly characterized by ki67% positivity, which must be less than 20%. For lung NETs, the terms typical and atypical carcinoids were used for G1 and G2, respectively. The differentiation of LNETs is not solely determined by ki67% positivity, but rather on the basis of mitotic count per 2 mm^2^, with a maximum threshold of 10 mitoses. However, it is essential to recognize that the classification of lung NENs incorporates not only the mitotic rate but also the presence and extent of necrosis. These tumors exhibit certain common phenotypic characteristics, including rosette formation, solid nesting architecture, and trabeculae, as well as the expression of neuroendocrine markers such as chromogranin A, synaptophysin, and CD56/NCAM. However, they also possess distinct features such as the microenvironment in which they grow, the molecular mutations they harbor, their response to various therapeutic regimens, and their biological behavior. Recognizing the similarities and differences between these tumors is crucial for developing more effective treatment strategies and identifying novel therapeutic options tailored to the specific characteristics of each group.

## 2. Methods

For this narrative review, a comprehensive literature search was performed to gather evidence on the treatment and characteristics of LNETs and GEP-NETs. The databases searched included MEDLINE/PubMed, and the search was concluded on 30 October 2023. Our search strategy was designed to encompass a broad range of terms relevant to our study objectives, including “lung neuroendocrine tumors”, “carcinoid”, “gastroenteropancreatic neuroendocrine tumors”, “EGFR”, “immunotherapy”, “chemotherapy”, “targeted therapy”, and “clinical trials”. We excluded articles not written in English to maintain consistency in the data analysis. The scope of our review deliberately omitted studies that focused on local therapeutic approaches for both LNETs and GEP-NETs, aiming to concentrate on systemic treatment. The selection of articles for inclusion was meticulously performed by the authors, prioritizing the most pertinent and recent publications that provided insights into the distinctions and similarities between LNETs and GEP-NETs, with particular emphasis on therapeutic outcomes and molecular characteristics.

## 3. Delineating the Diversity: Dissecting the Multifaceted Differences between Lung NETs and GEP-NETs

### 3.1. Genetic Alterations

Two studies have yielded essential insights into the molecular changes that occur in lung neuroendocrine tumors. Fernandez-Cuesta et al. conducted a study to investigate genetic mutations in 69 carcinoids through RNA sequencing (RNAseq) and 44 tumor-normal pairs through whole-genome sequencing or whole-exome sequencing (WES) in typical and atypical carcinoids. The results revealed that mutations in TP53 and RB1 occurred infrequently, and mutations in chromatin-remodeling genes were identified in approximately 51.1% of the analyzed samples. Alterations have been found in 40% of histone modifier genes, such as MEN1, PSIP1, and ARID1A, with no genetic segregation observed between typical and atypical carcinoids [7]. 

Asiedu et al. used gene expression and high-density single-nucleotide polymorphism (SNP) arrays to evaluate the classification of LNETs based on differential gene expression and copy number variation (CNV). The clustering of differentially expressed genes failed to differentiate between typical and atypical carcinoids, and no correlations were observed between clinical outcomes and gene expression in carcinoid tumors. Analysis revealed significant mutations in chromatin remodeling genes: DPF1, RNF212, and TAPBP. The most frequently mutated genes were ATP1A2, CNNM1, MACF1, RAB38, NF1, RAD51C, TAF1L, EPHB2, POLR3B, and AGFG1. Pathway analysis of differentially expressed genes with CNV changes identified the involvement of the NF-kB and MAPK/ERK signaling pathways [8].

In contrast, in a study of 724 GEP-NENs, including 335 low-grade cases, Puccini et al. discovered significant variations in genes that were most frequently mutated in pancreatic and low-grade GI NETs. Specifically, MEN1, ATRX, FOXO3, and PTEN were identified as the most frequently mutated genes in pancreatic NETs, whereas low-grade GI-NETs exhibited a higher, albeit not statistically significant, mutation rate in the APC gene (1.6% vs. 0%, *p* = 0.211) [9]. 

The gene expression profiles also differ markedly between LNETs and GEP-NETs. The research conducted by Alcala et al. identified the molecular clusters A1, A2, and B in LNETs, which have significant differences in gene expression. Specifically, carcinoids in cluster A1 exhibited high expression of ASCL1 and DLL3, whereas those in A2 showed the downregulation of SLIT1 and ROBO1 expression. Carcinoids in cluster B are characterized by high levels of ANGPTL3 and ERBB4, along with very low levels of OTP and NKX2-1 [10]. Furthermore, Miyanaga et al. uncovered notable upregulation in genes such as DENND1B and GRID1 in LNETs, providing insights into their molecular dynamics. Michele Simbolo and colleagues’ work on carcinoids reveals differential gene expression, with atypical carcinoids (AC) showing increases in genes such as TERT and SDHA, and typical carcinoids (TC) showing a loss in MEN1 [11,12].

GEP-NETs also displayed distinctive gene expression patterns, notably high levels of CHGA and CHGB. In small intestinal NETs (SiNETs), there is a marked increase in the expression of NEUROD1 and FOXA1, whereas pancreatic NETs (pNETs) are characterized by elevated levels of PDX1, PAX6, MAFA, NKX6-1, and RXRG [13]. Further insights include the detection of TUBB3 expression, MGMT methylation, and changes in TOP2A, PGP, PR, EGFR, and ER [9]. Gene expression studies have also identified specific subgroups of GEP-NETs that are associated with hypoxia and HIF signaling pathways [14].

The diversity of mutations, chromosomal alterations, and gene expression differences (as shown in Table 1) not only emphasizes the disparities between LNETs and GEP-NETs but also indicates promising avenues for targeted therapeutic interventions.

### 3.2. The Role of Epidermal Growth Factor Receptor

The epidermal growth factor receptor (EGFR) is a critical regulator of epithelial tissue development and maintenance, and its activation has been implicated in cancer progression. EGFR is a key driver of tumor growth in certain types of cancers, such as lung cancer and glioblastoma, and is also present in neuroendocrine tumors, exhibiting distinct expression patterns in GEP-NETs and lung NETs [15].

Papouchado et al. found that 91% of small intestine neuroendocrine tumors displayed positive immunohistochemical results for EGFR compared to only 25% of pancreatic neuroendocrine tumors. In these instances, EGFR was predominantly located in the cytoplasm, with only a minimal presence at the cell membrane. Additionally, immunohistochemical detection of EGFR is confined to focal localization rather than diffuse distribution [16,17]. In contrast, in pulmonary neuroendocrine tumors, EGFR is prominently displayed on the cell membrane in approximately 48% of cases and is typically expressed at high levels [18]. Elevated EGFR expression was more prominent in lung carcinoid tumors than in carcinomas, and upregulated EGFR expression was significantly associated with lower IASLC-Grade (*p* = 0.0005). Interestingly, when primary tumors and metastatic foci were analyzed by FISH for an elevated mean EGFR copy number, there was heterogeneity in test positivity, with 30% of cases having an elevated mean EGFR copy number in the primary focus but not in the metastatic lesion [19].

Despite the absence of detectable targeted mutations in the EGFR kinase domain in both GEP-NETs and lung carcinoids, preclinical data from a cancer cell series of LNETs suggested that the combination of Erlotinib and Everolimus may have synergistic effects on the inhibition of the EGFR/AKT/mTOR axis. Further in vivo and clinical investigations of the combined inhibition are warranted [20,21].

### 3.3. ASCL1 and DLL3 

The transcription factor ASCL1 (Achaete-scute homologue 1) is critical for neuroendocrine differentiation, as evidenced by the findings of a study that showed that mice deficient in ASCL1 did not exhibit pulmonary neuroendocrine cells [22]. Furthermore, ASCL1 regulates the expression of DDL3, a Notch ligand that is highly expressed in SCLC and other neuroendocrine tumors but is minimally expressed in normal tissues [23].

ASCL1 is highly frequent in Small Cell Lung Cancer (SCLC), occurring in approximately 70% of cases and can be detected immunohistochemically [24]. Its presence is associated with the upregulation of several genes and expression of DLL3 receptors [25,26]. A subtype with high ASCL1 and DLL3 expression has been identified in type I LCNEC, and similar findings have been reported by Alcala et al. for Carcinoid A1 [10,27]. Targeting DLL3 receptors is a promising area of research for many targeted therapies being tested in clinical trials for neuroendocrine tumors [28,29,30]. However, DLL3 expression is significantly more frequent in aggressive neuroendocrine neoplasms than in typical and atypical carcinoids [31,32].

Notch has been suggested to function as a tumor suppressor in ileal NETs/carcinoids, because its expression is low or absent in these tumors. Notch signaling in non-tumorigenic cells triggers a series of events that ultimately lead to ASCL1 protein inhibition. ASCL1 is overexpressed in ileal NETs and in vitro experiments have shown that transient overexpression of Notch1 in carcinoid cell lines can reverse ASCL1 overexpression, indicating that Notch1 activation may be a potential therapeutic strategy [33]. Studies have shown that increased ASCL1 expression in gastrointestinal-derived neuroendocrine neoplasms is detected mainly in high-malignancy carcinomas rather than in slower-progressing GEP-NETs, and at a lower frequency compared to lung neuroendocrine carcinomas [34,35]. In a study of 47 patients with GEPNENs, including both NETs and NECs, immunohistochemical detection of DLL3 in formalin-fixed, paraffin-embedded (FFPE) samples was absent in all well-differentiated GEP-NETs and high-grade features (G3 NET) and was present in 76.9% of poorly differentiated NECs (G3 NEC) [36].

### 3.4. PDL1 and Immune Response

In a recent study, formalin-fixed paraffin-embedded (FFPE) samples from 168 patients with pulmonary neuroendocrine tumors were analyzed. A 1% cut-off value was employed, and 5% of typical carcinoids were deemed positive for programmed death ligand 1 (PD-L1) expression. Atypical carcinoids were negative for PD-L1 expression. The expression of PD-L1 was observed to be significantly associated with mediastinal lymph node metastasis at the time of diagnosis, as well as the overall metastatic potential of the tumor. Notably, typical carcinoids showed a slightly lower CD8+ T cell density than atypical carcinoids did. However, this difference was not statistically significant [37]. Tsuruoka et al. conducted an immunohistochemical analysis of 227 lung NET patients, utilizing the E1L3N PD-L1 antibody clone for tissue microarray samples. Their research highlighted that PD-L1 expression was present in 10.4% of large-cell neuroendocrine carcinoma (LCNEC) cases and 5.8% of small-cell lung cancer (SCLC) cases, whereas it was absent in typical and atypical carcinoid tumors [38].

A study conducted by Rösner et al. revealed that PD-L1 expression in lung NENS is positively correlated with tumor grade, a higher Ki-67 index, and enhanced CXCR4 expression, while displaying an inverse association with somatostatin receptor 1 and chromogranin, suggesting that PD-L1 expression is not only prevalent in lung NENs but also escalates with tumor malignancy and might be linked to poorer patient outcomes [39].

In GEP-NETs, the expression of PDL1 is infrequent. In the archival tissue of 64 well-differentiated small intestine NETs and 31 pancreatic neuroendocrine tumors (pNETs), no cases of small intestine NETs exhibited tumoral PD-L1 expression, whereas only 7.4% of pancreatic NETs showed such expression. Additionally, high CD8 intratumoral detection was observed in 3% of pancreatic NETs, whereas no such detection was found in small intestine NETs [40]. 

In a study conducted by Cavalcanti et al., it was discovered that PD-L1 expression has a strong correlation with higher-grade GEP-NENs, particularly grade 3 (G3). This suggests that there is a complex interaction between tumor aggressiveness and immune evasion mechanisms. Interestingly, the expression of PD-L1 was not affected by sex, primary tumor site, or lymph node status but was significantly associated with tumor grade. This highlights its potential as a biomarker for tumor grading. The study found that, as the tumor grade progressed from G1 to G3, there was a notable increase in PD-L1 expression in both tumor cells and immune-infiltrating cells [41].

The microenvironments of typical and atypical carcinoids do not exhibit an immunologically sterile profile. According to Alcala et al., dendritic cells were detected in the majority of carcinoids (60%), whereas the presence of alveolar macrophages in large or small numbers did not seem to be associated with patient prognosis [10]. Single-cell analysis revealed significant intratumoral heterogeneity in these tumors, with a variety of immune cells, including conventional T cells, CD8+ T cells, NK cells, B cells, and plasma cells, present in the microenvironment. The detection rate of lymphoid cell types was similar to that of healthy tissues, and exhaustion signature scores were low. In contrast, populations of monocytes, macrophages, and mast cells were found at different concentrations compared to those in normal tissues, with a prominent washout of monocytes [42]. It has been observed that the expression of IFNγ-associated genes and intratumoral T-cell infiltration are low in both NET G1/G2 and NET G3/NEC. While neuroendocrine carcinomas (NECs) exhibit hot immune microenvironments with an abundance of tumor-infiltrating lymphocytes (TILs), neuroendocrine tumors (NETs) possess a cold immune microenvironment with fewer TILs [43]. Among the comparatively well-differentiated GEP-NETs, pancreatic NETs exhibited higher TILs, PD-1, and PD-L1 expression levels than non-pancreatic NENs. Among SINENs, duodenal NENs demonstrate higher immune infiltration than jejunal or ileal NENs [44].

### 3.5. Spread through Air Spaces (STAS)

STAS is defined as the presence of micropapillary (MP) clusters, solid nests, or single cells extending beyond the primary tumor into air spaces. Currently, STAS is widely acknowledged as an invasive adenocarcinoma pattern. There is a debate on whether STAS is a real phenomenon or an artifact. Although some evidence suggests that it is an artifact created during tissue processing, many studies have shown that it is a significant risk factor for recurrence. Standardization in diagnosing STAS is lacking and more studies are required to reach a consensus.

In a study by Altinay et al., STAS was present in 48% of patients with atypical carcinoids as opposed to 20.5% of patients with typical carcinoids. This finding was later corroborated by a study by Chae et al., who discovered STAS in 22% and 50% of patients with typical and atypical carcinoids, respectively [45,46].

In a study conducted by Aly et al., 487 patients with typical carcinoids, atypical carcinoids, large-cell neuroendocrine carcinomas, and small-cell lung carcinomas were evaluated for STAS. Multivariate analysis stratified by stage indicated that STAS was significantly associated with a higher risk of recurrence and death in the overall cohort and in the AC, LCNEC, and SCLC subgroups. However, owing to the limited number of recurrences and deaths in the typical carcinoid cohort, prognostic analysis could not be performed [47].

STAS, along with EGFR localization and the specific genetic alterations highlighted, add to a series of features that illuminate the multifaceted differences between LNETs and GEP-NETs, as shown in Table 2. These elements collectively underscore complex disparities in genetic, molecular, and pathological aspects, providing a comprehensive overview of their distinct biological landscapes.

## 4. Navigating Therapeutic Strategies: Efficacy in Treating Lung NETs and GEP-NETs

### 4.1. Somatostatin Analogues 

The available data on the effectiveness of somatostatin analogs in the treatment of GEP-NETs are more abundant than those for LNETs. Although there has been no direct comparison between the two groups, certain inferences can be drawn from the combined data that currently exist. In the Clarinet study, lanreotide demonstrated a statistically significant improvement in progression-free survival (PFS) for both G1 and G2 GEP-NETs. It is worth noting that up to 30% of G2 patients were included in the study, and the hazard ratio (HR) for PFS in G2 NETs was 0.45 (0.22–0.91), similar to the HR of 0.43 (0.25–0.74) in G1. The median PFS for all GEP-NETs was also statistically significant, with an HR of 0.47 (95% CI, 0.30 to 0.73). In contrast, the SPINET trial, which assessed lanreotide in typical and atypical lung carcinoids, did not show a statistically significant improvement in PFS with an HR of 0.90 (95% CI, 0.46, 1.88; *p* = 0.769); however, it is essential to note that lanreotide’s inability to significantly extend PFS may be attributed to the low patient accrual in the trial, which suggests a lower statistical power. Moreover, a differential treatment benefit between typical and atypical carcinoids was not demonstrated because of the limited number of progression-free survival (PFS) events, hindering reliable interaction analysis between histology (typical or atypical carcinoid) and the efficacy of lanreotide. Instead, the trial highlighted the prognostic effect of histology on PFS, with patients having typical carcinoids showing a better prognosis than those with atypical carcinoids [48].

Newer data from the utilization of Gallium-68-DOTATATE (GaTATE) in LNETs have demonstrated significant interpatient heterogeneity of somatostatin receptor expression in both typical carcinoids (TC) and atypical carcinoids (AC) [49]. This interpatient heterogeneity should be considered when interpreting the outcomes of the SPINET trial to ensure accurate and reliable results.

Considering the distinctions highlighted and informed by data from retrospective studies, a recent publication by experts in neuroendocrine tumor management recommended the application of somatostatin analogs (SSAs) exclusively for low-grade metastatic lung neuroendocrine tumors (LNETs). While current guidelines position SSAs as a viable alternative treatment for advanced LNETs, it is important to note that their formal approval for pulmonary neuroendocrine tumors remains pending [50].

### 4.2. Peptide Receptor Radionuclide Therapy (PRRT) in Lung NETs

The NETTER 1 study demonstrated the significant benefits of PRRT in GEP-NETs and introduced the potential for therapeutic effects in neuroendocrine tumors [2]. The ability to target lesions due to somatostatin receptor expression offers the potential for targeted treatment of multiple foci, whereas the effect of treatment on adjacent clones within the lesion via the cross-fire effect may help treat heterogeneous metastatic foci [51]. However, it should be noted that this study did not include LNETs and its efficacy in these tumors has not been confirmed. Although metastatic lung carcinoids often do not express somatostatin receptors homogeneously or at the same level as GEP-NETs, retrospective studies have suggested that PRRT may be an effective treatment for a limited number of patients with metastatic lung NETs with high and homogeneous SSR expression [49,52,53]. It is important to note that immunohistochemical detection of somatostatin receptors in pulmonary NETs may not always match the detection by 68Ga-DOTANOC-positron emission tomography, suggesting that negative immunohistochemistry for SSR should not discourage clinicians from requesting 68Ga-DOTANOC-positron emission tomography for staging lung NETs [54].

### 4.3. Everolimus

The mammalian target of rapamycin (mTOR) plays a pivotal integrative function in numerous cellular processes and serves as a receptor for extracellular stimuli derived from energy levels, nutrient availability, growth factors, oxygen supply, and stress. Its prominent role in the development and progression of NETs has been extensively documented in both preclinical research and late-stage clinical trials [55].

The mTOR kinase inhibitor everolimus, which is currently the only approved targeted therapy for pulmonary neuroendocrine tumors, exhibited comparable benefits in both GEP-NETs and pulmonary NETs, as demonstrated in the RADIANT 4 study. Furthermore, the significance of these benefits persisted regardless of the prior treatment received by the patients, and real-world data have confirmed the similar efficacy of the drug for both types of NETs [56,57,58]. The subgroup analysis revealed no meaningful discrepancies in efficacy between typical and atypical carcinoids, suggesting comparable advantages from treatment across these histologies. Although the incidence of grade ≥ 3 toxicity increases significantly in patients pretreated with chemotherapy or PRRT, administering the drug early in the treatment line series has been shown to significantly reduce the incidence of adverse side effects. Additionally, a reduction in dosage did not appear to have a statistically significant effect on efficacy, although a numerical difference was observed [59].

### 4.4. Chemotherapy

#### 4.4.1. 5-FU or Capecitabine-Based Regimens

The use of 5-FU in gastrointestinal neuroendocrine tumors is based on its long-standing use as the backbone of chemotherapy in gastrointestinal adenocarcinomas, as both tumor types share a common microenvironment, despite differences in genetic mutations and pathological pathways. However, 5-FU has not been proven effective in the treatment of non-small cell lung cancer and small cell neuroendocrine carcinoma (SCLC); thus, it is not used in clinical practice [60,61]. Nevertheless, some studies have suggested that its use for lung NETs may be beneficial in certain cases. For instance, retrospective data showed that 20% of patients with pulmonary NETs responded to treatment with either FOFLOX or GEMOX, and the combination of FOLFOX with bevacizumab has been demonstrated to be effective for both GEP-NETs and Lung NETs, although the number of patients with lung NETs was small in this study, and maintenance bevacizumab treatment appeared to provide a significant survival benefit for these patients [62,63,64].

The efficacy of XELOX was evaluated in a retrospective study of patients with pulmonary GEPNETs. The study comprised five individuals with lung NETs, three of whom demonstrated a response to treatment according to the I.T.M.O. criteria, which assess tumor growth, symptom presence/severity, and marker behavior separately rather than the RECIST criteria [65]. Owing to the limited number of patients with pulmonary carcinoids in the aforementioned studies, it is essential to conduct large prospective multicenter trials to determine the efficacy of 5-FU-based therapies for the treatment of pulmonary neuroendocrine tumors. FOLFIRI, a commonly used regimen for adenocarcinomas of the gastrointestinal tract, has also been administered to patients with neuroendocrine neoplasms, particularly to those with neuroendocrine carcinomas (NECs) [66]. Currently, limited data are available on the efficacy of this treatment for pulmonary neuroendocrine tumors, and its value in clinical practice has yet to be validated.

#### 4.4.2. Streptozocin-Based Regimens

Streptozocin (STZ) is an approved drug for the treatment of metastatic pancreatic neuroendocrine tumors. Although it is commonly used off-label for the treatment of gastrointestinal neuroendocrine tumors, its efficacy in lung neoplasms has not been demonstrated. However, it has also been tested for pulmonary carcinoids. In a clinical trial, patients with both GEP-NETs and Lung NETs were tested with a combination of STZ and either cyclophosphamide or 5-fluorouracil, revealing a difference in the response between the two groups. The combination of STZ with cyclophosphamide showed an objective response rate (ORR) of 0% in patients with pulmonary NETs and 37% in patients with GEP-NETs, whereas the combination of STZ with 5-FU resulted in a significantly higher ORR in pulmonary NETs, although it was still lower than that in GEP-NETs (29% vs. 44%) [67]. 

#### 4.4.3. Temozolomide Plus Capecitabine

Combination therapy with capecitabine and temozolomide, commonly known as CAPTEM, has shown significant efficacy in patients with pancreatic neuroendocrine tumors. Reports indicate that the objective response rates for this treatment approach vary between 33% and 70%, while the median progression-free survival spans from 18 to 22.7 months [68,69,70]. A retrospective study assessed the efficacy of temozolomide monotherapy in 31 patients with metastatic lung neuroendocrine tumors, achieving a response rate (RR) of 14% and median progression-free survival (PFS) of 5.3 to 13 months [71]. Another study evaluated the effectiveness of combination therapy of temozolomide and capecitabine in 20 patients with lung NETs, demonstrating an RR of 30% and a median PFS of 13 months [72]. Despite the absence of direct comparative studies on the efficacy of TEMCAP between LNETs and GEP-NETs, the evidence available suggests a comparatively lower efficacy of TEMCAP in treating lung carcinoids as opposed to GEP-NETs. A recent multicenter trial indicated that the origin of the primary tumor did not exhibit a statistically significant influence on the efficacy of combination therapy; however, the line of treatment in which it was employed displayed a notable impact [73].

### 4.5. Immunotherapy in LNETs and GEP-NETs

Immunotherapy has significantly transformed the treatment landscape of lung cancer, offering substantial improvements in patient outcomes. However, its effectiveness in combating gastrointestinal cancer is limited. Notably, certain subsets of patients, particularly those with mismatch repair (MMR)-deficient tumors or high tumor mutational burden (TMB-high), have shown enhanced benefits from immunotherapy. Studies have investigated the effectiveness of immunotherapy in LNETs and GEP-NETs with varying results (Table 3).

The Dune study was conducted in a multicenter setting to evaluate the efficacy of durvalumab in combination with tremelimumab for various types of neuroendocrine tumors, including lung neuroendocrine tumors. Patients with typical and atypical carcinoids (Lung NETs) demonstrated the highest overall response rate (ORR), with no statistically significant difference in progression-free survival (PFS) between the Lung NETs and other groups, such as gastrointestinal and pancreatic NETs. However, in terms of overall survival, patients with Lung NETs experienced a greater benefit than those in other study groups [76].

Pembrolizumab has been investigated in neuroendocrine neoplasms in the KEY-NOTE-028 and KEYNOTE-158 clinical trials using varying dosing schedules [74,75]. Despite this, both studies enrolled patients with neuroendocrine neoplasms of lung and gastrointestinal origins. In both trials, pembrolizumab was used solely as monotherapy without being combined with other treatments. The observed overall response rate (ORR) suggested a higher effectiveness for treating lung neuroendocrine tumors than gastrointestinal or pancreatic NETs. However, it is important to interpret these findings with caution due to the absence of head-to-head comparative studies. Thus, while preliminary data indicate a potential advantage for LNETs, further research is needed to conclusively determine the relative efficacy of immunotherapy across different NET types. The treatment also had a good toxicity profile, with rates similar to those reported in clinical studies on other neoplasms.

Spartalizumab has been evaluated as a monotherapy for the treatment of neuroendocrine tumors, demonstrating varying levels of efficacy depending on the origin of the tumor. Specifically, the overall response rate for lung carcinoids was 16.7%, whereas that for GEPNET was 3%. These findings indicate that immune responses differ between the two groups [77].

Toripalimab is another immune checkpoint inhibitor that has demonstrated objective response rates (ORR) in individuals with neuroendocrine tumors. In this study, patients were classified into three distinct groups based on the origin of their neuroendocrine tumors: pancreatic NENs, gastrointestinal NENs excluding those from the pancreas (ex-pancreatic GI NENs), and NENs arising outside the gastrointestinal system (non-GI NENs). The overall response rates (ORRs) observed varied significantly with tissue origin, with ex-pancreatic GI NENs demonstrating an ORR of 13.0%, pancreatic NENs showing 22.2%, and non-GI NENs achieving the highest at 37.5%. Furthermore, the response rates for poorly and well-differentiated NETs were 18.7% and 25%, respectively [79].

In the NET-001 and NET-002 trials, 21 patients with GEPNET and six patients with neuroendocrine lung carcinomas were administered avelumab monotherapy, although patients with typical carcinoids were excluded from the study. No objective responses were observed in either of the groups. Nevertheless, stable disease was achieved in 33% of patients, and the disease control rate at six months was 21% [78]. In the DART SWOG 1609 study, which examined the combination of Nivolumab with Ipilimumab in both well-differentiated NETs and NECs, no objective responses were observed in NETs regardless of their origin [80].

In the NCT03728361 study, the efficacy of nivolumab in combination with temozolomide was evaluated in NETs and neuroendocrine carcinomas originating from various organs, and the study confirmed that patients with LNETs showed a higher response rate than those with NETs from other organs (lung vs. others, *p* = 0.020). The median progression-free survival (PFS) reached 8.8 months, with a 95% confidence interval (CI) of 3.9 to 11.1 months, while the median overall survival (OS) was recorded at 32.3 months, with a 95% CI ranging from 20.7 months to an upper limit not yet reached. The study reported a response rate of 32% across all NETs, with a notably higher response rate of 64% among patients with LNETs. Verified responses were specifically recorded in cases of lung and pancreatic cancer [81].

The clinical trial NCT03074513 was conducted to evaluate the efficacy of atezoli-zumab in combination with bevacizumab in patients with advanced, progressive grade 1–2 neuroendocrine tumors. A total of 40 patients were enrolled, including 20 with pancreatic neuroendocrine tumors and 20 with extrapancreatic neuroendocrine tumors (epNETs), and five of them had LNETs. The results showed that 20% of the patients with pNETs and 20% of those with epNETs achieved an objective response. Additionally, this study found that PD-L1 expression in greater than 1% of tumor cells by immunohistochemistry may be associated with efficacy [82].

## 5. Discussion

The growing recognition of specific genetic and molecular characteristics that distinguish LNETs from GEP-NETs highlights the need for a precision medicine approach tailored to the individual characteristics of each tumor type. This tailored approach is essential for improving diagnostic accuracy and therapeutic effectiveness. The variation in their responses to treatments such as somatostatin analogs, peptide receptor radionuclide therapy (PRRT), chemotherapy, and immunotherapy also underscores the biological differences between the two tumor types and argues against a universal treatment approach.

Additionally, the tumor microenvironment of LNETs and GEP-NETs varies, with significant differences in immune system cell concentration and composition. These discrepancies are particularly important in the context of immunotherapy, where LNETs generally display higher response rates than GEP-NETs, except for pancreatic NETs. Moreover, the presence of spread through air spaces (STAS) in LNETs distinguishes them from GEP-NETs and highlights the need for specific translational approaches to understand the unique pathophysiological mechanisms that contribute to the carcinogenesis of these diseases.

## 6. Future Directions and Conclusions

In the future, it is essential to recognize that neuroendocrine tumors exhibit distinct characteristics based on their origin, necessitating a departure from conventional classification and treatment strategies. This nuanced understanding can inform the design of more effective clinical trials and therapeutic strategies, particularly as we explore the potential of targeting specific molecular pathways, such as EGFR and DLL3, which present promising avenues for innovative treatments. The differential expression of EGFR in pulmonary NETs, despite the absence of detectable targetable mutations, and the emerging role of DLL3 as a therapeutic target in advanced and metastatic neuro-endocrine tumors, highlights the evolving landscape of treatment options. Furthermore, incorporating STAS into patient assessment for lung NETs could enhance both risk evaluation and therapeutic decision making.

The recognition of neuroendocrine tumors’ (NETs) diverse characteristics and treatment responses is pivotal, emphasizing the imperative for personalized management strategies. This review highlights the critical need to differentiate between lung neuroendocrine tumors and gastroenteropancreatic neuroendocrine tumors as well as the necessity to further subcategorize GEP-NETs into pancreatic and non-pancreatic tumors. Presented evidence, supported by the literature, supports a customized approach to NET treatment, considering each tumor’s unique origin, genetics, microenvironment, and morpho-biological features. Such a refined understanding facilitates the crafting of tailored treatment protocols to enhance patient outcomes for these intricate diseases.

To augment the efficacy of combination therapies, it is essential to design clinical trials that acknowledge the heterogeneity of LNETs, pancreatic NETs, and gastrointestinal NETs. By stratifying NETs according to their specific origins and molecular profiles, we can pave the way for innovative combination treatments that optimize their efficacy while minimizing adverse effects. Embracing an individualized treatment paradigm rooted in an In-depth understanding of the distinct characteristics of LNETs, GI-NETs, and pNETs is crucial for propelling neuroendocrine tumor research and improving patient care.

## Figures and Tables

**Table 1:** Comparative genetic and molecular landscape of lung neuroendocrine tumors (LNETs) and gastroenteropancreatic neuroendocrine neoplasms (GEP-NENs).

Study	NENs	Most Common Mutations	Chromosomal Alterations	Changes in Gene Expression
Fernandez-Cuesta et al. [7]	LNETs	Chromatin-Remodeling Genes: MEN1, PSIP1, and ARID1A Other mutations: EIF1AX, SEC31A, WDR26, and HERC2	No significant focal copy number alterations reported One case of chromothripsis in chromosomes 3, 12, and 13	No changes in gene expression unrelated to mutations or chromosomal alterations.
Asiedu et al. [8]	LNETs	ATP1A2, CNNM1, MACF1, RAB38, NF1, RAD51C, TAF1L, EPHB2, POLR3B, and AGFG1) Other mutations: TMEM41B, DEFB127, WDYHV1, and TBPL1	Deregulation of NF-κB and MAPK/ERK pathways based on CNV analysis	Not directly addressed
Alcala et al. [10]	LNETs	MEN1, ARID1A, and EIF1AX, ATM, PSIP1, and ROBO1 Alterations in covalent histone modifiers and SWI/SNF complex	Chromothripsis involving chromosomes 11 and 20 Inter chromosomic rearrangement between genes MEN1 and SOX6	High expression of ASCL1 and DLL3 in Carcinoids A1 Downregulation of SLIT1 and ROBO1 in Carcinoids A2 High levels of ANGPTL3, ERBB4, and very low levels of OTP and NKX2-1 in Carcinoids B
Miyanaga et al. [11]	LNETs	MUC6, SPTA1	TRIB2-PRKCE fusion	Upregulation of DENND1B, GRID1, CLMN, DENND1B, NRP1, SEL1L3, C5orf13, TNFRSF21, TES, STK39, MTHFD2, OPN3, MET, and HIST1H3C
Simbolo et al. [12]	LNETs	MEN1, KMT2D, RB1, and TERT	Mutations in histone modifiers and members of the SWI-SNF complexes	AC: gains in TERT, SDHA, RICTOR, PIK3CA, MYCL, and SRC TC: loss of MEN1
Hoffman et al. [13]	GEP-NETs	MEN1, VHL, TSC1/2 ATRX in pNETs	Chromosomal loss, telomere alterations: Identified as potential regulators of GEP-NET development	CHGA and CHGB NEUROD1 and FOXA1 (SiNETs) PDX1, PAX6, MAFA, NKX6-1, and RXRG (pNETs)
Puccini et al. [9]	GEP-NETs	Low-Grade Tumors: ATRX (13%), ARID1A (10%), MEN1 (10%) High-Grade Tumors: TP53 (51%), KRAS (30%), APC (27%), ARID1A (23%)	Not directly addressed	TUBB3, MGMT methylation, TOP2A, PGP, PR, EGFR, ER expression
Scarpa et al. [14]	GEP-NETs	MUTYH, CHEK2, BRCA2. MEN1, VHL, PTEN, DEPDC5, TSC1, TSC2	Gene fusions, especially involving EWSR1 with BEND2 and FLI1, and chromothripsis in 9% of tumors Losses in MEN1 and CDKN2A, gains in genes like PSPN and ULK1	Gene expression analyses identified subgroups of tumors associated with hypoxia and HIF signalling

NENs: neuroendocrine neoplasms, LNETs: Lung neuroendocrine tumors, GEP-NETs: gastroenteropancreatic neuroendocrine tumors, pNETs: pancreatic neuroendocrine tumors.

**Table 2:** Comparison of molecular characteristics between LNETs and GEP-NETs: This table delineates the key genetic alterations, signaling pathways involved, and potential therapeutic targets identified in LNETs versus GEP-NETs.

Characteristic	LNETs	GEP-NETs
Frequently mutated genes	ATP1A2, CNNM1, and MACF1	ATRX, ARID1A, and MEN1
Pathway involved	MAPK/ERK and NF-kB	PI3K/Akt/mTOR INK4a/ARF and RB1
EGFR	Detected in cell membrane Overexpressed in 48% of LNETs	Detected in cytoplasm focally
Tumor immune microenvironment (TIME)	Heterogeneous	pNENs express higher TILs, PD-1 compared to other GEP-NETs
Spread Through Air Spaces (STAS)	Described	
DLL3	Overexpressed in Carcinoids A1	Absent in low-grade GEP-NETs

LNETs: Lung neuroendocrine tumors, GEP-NETs: gastroenteropancreatic neuroendocrine tumors, pNETs: pancreatic neuroendocrine tumors, EGFR: epidermal growth factor receptor, Carcinoid A1: a molecular cluster defined by Alcala et al., TILs: tumor-infiltrating lymphocytes.

**Table 3:** Overview of immune checkpoint inhibitor trials in neuroendocrine tumors across different organs.

Drug	Line	ORR LNETs	ORR GI NETs	ORR pNETs
Pembrolzumab [74]	≥2	NR	2%	7.5%
Pembrolzumab [75]	≥2	12.0%	NI	6.3%
Durvalumab plus tremelimumab [76]	≥2	11.1%	0.0%	6.3%
Spartalizumab [77]	≥2	16.7%	3.1%	3.0%
Avelumab [78]	≥1	Νo objective responses in NETs		
Toripalimab [79]	≥2	NR	13.0%	22.2%
Ipilimumab plus nivolumab [80]	≥1	Νo objective responses in NETs		
Nivolumab plus Temozolomide [81]	≥1	64%	NR	67%
Atezolizumab plus Bevacizumab [82]	≥3	NR	NR	20%

ORR: Overall Response Rate, LNETs: Lung neuroendocrine tumors, GI NETs: gastrointestinal neuroendocrine tumors, pNETs: pancreatic neuroendocrine tumors, NR: not reported, NI: not included.

## Data Availability

No new data were created or analyzed in this study. Data sharing is not applicable to this study.
